# Fraxin Prevents Chemically Induced Hepatotoxicity by Reducing Oxidative Stress

**DOI:** 10.3390/molecules22040587

**Published:** 2017-04-06

**Authors:** Bo Yoon Chang, Young Suk Jung, Chi-Su Yoon, Jun Seok Oh, Jae Heoi Hong, Youn-Chul Kim, Sung Yeon Kim

**Affiliations:** 1 Institute of Pharmaceutical Research and Development, College of Pharmacy, Wonkwang University, Iksan, Jeonbuk 54538, Korea; oama611@nate.com (B.Y.C.); ycs1991@naver.com (C.-S.Y.); wku114@naver.com (Y.-C.K.); 2 College of Pharmacy, Pusan National University, San 30, Jangjeon-dong, Busan 46241, Korea; youngjung@pusan.ac.kr; 3 Dongbu Eastern Herbal Medicine Agricultural Association Corporation, Yeosunro 1679, Sunchun-si, Jeonnam 58019, Korea; 5811684@daum.net (J.S.O.); 5811684@naver.com (J.H.H.)

**Keywords:** antioxidant, *A. tegmentosum*, fraxin, hepatoprotective, HO-1, Nrf2

## Abstract

Fraxin isolated from *Acer tegmentosum* is reported to exert potent anti-oxidative stress action. However, pharmacological activities of fraxin remain to be elucidated. This study investigated the potential hepatoprotective effects of fraxin and the underlying signaling mechanism involved. Treatment with fraxin significantly lowered the serum levels of aspartate aminotransferase (AST) and alanine aminotransferase (ALT) in a CCl_4_-induced hepatotoxicity rat model. In the fraxin-treated group, glutathione (GSH) significantly increased, while the malondialdehyde (MDA) in the liver significantly decreased. Fraxin also showed radical-scavenging activity. Furthermore, it significantly reduced the t-BHP-induced cytotoxicity and production of reactive oxygen species (ROS) in Hep G2. Fraxin protected Hep G2 cells through Nrf2 pathway-dependent HO-1 expression. The results of this study indicate that fraxin shows potent hepatoprotective effects in vitro and in vivo, presumably through direct antioxidant activity and the Nrf2-mediated antioxidant enzyme system.

## 1. Introduction

Hepatic damage can be attributed to various factors, such as infections, autoimmune disorders, chemical agents, and excessive alcohol consumption [1,2,3,4]. Chemical-induced hepatotoxicity accounts for approximately half of the cases of acute liver failure. General mechanisms involved in chemical-induced liver injury include reactive metabolite formation, oxidative stress, and glutathione (GSH) depletion. Research in the field of free radical biology has shown that free radicals and oxidative stress play an important pathophysiological role in development and progression of liver diseases. The overproduction of free radicals and other reactive oxygen species (ROS) in liver cells causes oxidative stress of vital cellular macromolecules, such as lipids, proteins, and nucleic acids, leading to cell dysfunction and death. Oxidative stress is a key phenomenon in chronic diseases and hepatotoxicity induced by various chemicals [3,5]. Oxidative intermediates exert their toxic effect by destroying cellular defense mechanisms [6].

The human body is able to counteract oxidative stress by producing antioxidants, which are either naturally produced in situ or externally supplied through foods and/or supplements. Antioxidants can terminate chain reactions by removing free radical intermediates and inhibiting other oxidation reactions via oxidization of themselves [7].

Natural antioxidants can prevent free radical-mediated oxidative damage to cellular components by interacting at different levels in the pathophysiological pathway. Several plant extracts and their constituents have been found to possess hepatoprotective properties by improving the antioxidant status in vivo [2,8]. Therefore, plant-derived antioxidants may be particularly important in reducing the incidence of various liver disorders as well as other oxidative stress-related diseases.

*A. tegmentosum* (Aceraceae) is a deciduous tree found in Korea, Russia and China. In Korea, *A. tegmentosum* has been used in traditional medicine for treatment of hepatic disorders [9,10]. More than 20 components have been isolated from *A. tegmentosum*, including flavonoids (quercitrin, hyperin, myricitrin, (+)-catechin, gallocatechin, kaempferol-3-rhamnoside, and erigeside B), phenolic glycosides (salidroside, phenylethyl-*O*-β-d-xylopyranosyl-(1-2)-β-d-glucopyranoside, 3′-*O*-galloylsalidroside, 6′-*O*-galloylsalidroside), steroidal glycosides (β-sitosterol-3-*O*-β-d-glucopyranoside) and coumarins (fraxin and esculetin) [11,12].

**Figure 1:**
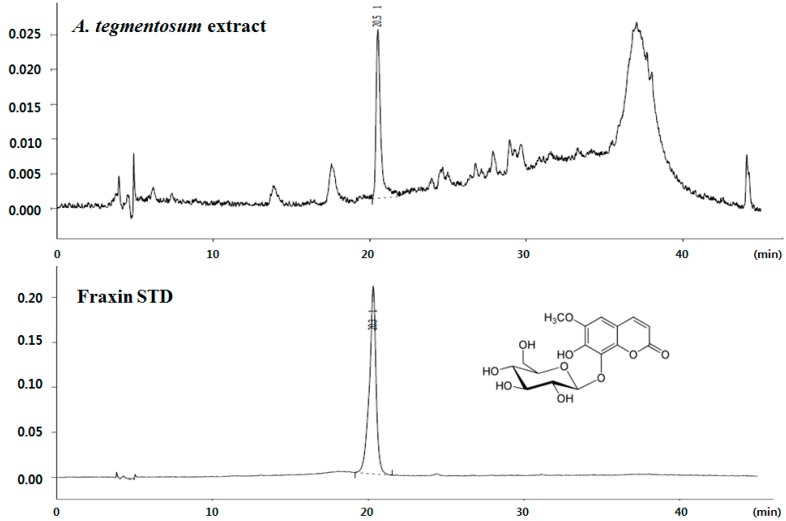
The structure and high-performance liquid chromatography (HPLC) chromatographic profile of fraxin in *A. tegmentosum* extract at 340 nm.

We directly compared the antioxidative effects of components, such as quercetin, hyperin, salidroside, exculetin, or fraxin. As a result, all of the components showed high antioxidative effects similar to positive control (50 μM vitamin C). The content of fraxin in *A. tegmentosum* extract was quantitated using high-performance liquid chromatography (HPLC). Results indicated that *A. tegmentosum* extract possessed 0.45 mg of fraxin per gram of extract (Figure 1).

Previous reports indicate that fraxin has antioxidant and low toxicity [13]. However, the mechanisms of the antioxidative and hepatoprotective activities of fraxin have not yet been studied. We hypothesized that the hepatoprotective activity of known *A. tegmentosum* constituents could be identified based on their antioxidant properties. On the basis of this, fraxin was selected as a potential hepatoprotective compound of *A. tegmentosum*. In the present study, we investigated the hepatoprotective effects of fraxin and the underlying signaling mechanism involved.

## 2. Materials and Methods

### 2.1. Experimental Animals and Design

Male Sprague–Dawley rats (weighing 160–170 g) were supplied by Orient Bio (Jeonbuk, Korea) and were fed a standard diet (Orient Bio, Jeonbuk, Korea) with access to tap water ad libitum. The study was approved by the Wonkwang University Animal Care Committee (WKU16-72).

Fraxin was purchased from Sigma Aldrich (St. Louis, MO, USA) and used without further purification. Doses were chosen to be in an effective and nontoxic range according to values derived from the literature or as determined in preliminary experiments. According to Wang et al., Fraxin was orally administered to mice at 500 mg/kg and no toxicity was observed [13]. The positive controls were gavaged with silymarin (50 to 100 mg/kg) in CCl_4_ induced hepatotoxicity rats [4,14,15]. We sought to evaluate the hepatoprotective effect of fraxin on the same dose as that of the positive control. Fraxin was administered by oral administration dissolved in D.D.W. Fraxin doses of 5, 10, and 50 mg/kg were applied to rats for five consecutive days. Acute liver injury was induced by a single administration of CCl_4_ (0.75 mL/kg, orally, diluted in corn oil) to rats 1 h after the final dose of the fraxin or silymarin was administered. The rats were euthanized 24 h later by exsanguination from the abdominal aorta under ether-induced anesthesia. Blood and liver tissue samples were collected for analysis.

### 2.2. Estimation of Biochemical Parameters

Serum from the collected blood samples was used for the determination of aspartate aminotransferase (AST) and alanine aminotransferase (ALT) levels according to the method described by Reitman and Frankel [16]. The substrates in the reaction are α-ketoglutaric acid and l-aspartate for AST, and α-ketoglutaric acid and l-alanine for ALT. The mixture was added with DNPH and was kept at room temperature. NaOH was added and the color development was read at 540 nm. Malondialdehyde (MDA), the end product of lipid peroxidation, was measured using a slightly modified thiobarbituric acid reactive substance assay [17]. Briefly, tissue homogenate was mixed with TBA solution (0.67%, *w*/*v*) solution. The mixture was then heated in a boiling water bath for 60 min. Afterwards butanol was added and centrifuged. The absorbance was measured at 532 nm with respect to the blank solution. The concentrations of GSH were determined based on GSH oxidation with DTNB (5,5′-dithiobis-2-nitrobenzoic acid), and the concentration of GSSG was determined enzymatically by glutathione reductase after inhibiting GSH oxidation with 2-vinylpyridine [18]. The optical density of the reaction product was read immediately at 412 nm on a spectrophotometer.

### 2.3. Histopathological Procedure

Liver tissues were placed in plastic cassettes and were immersed in 4% paraformaldehyde for 6 h. The fixed tissues were processed as described previously [19]. The degree of hepatocellular damage was evaluated by measuring the area of necrosis in liver sections stained with hematoxylin and eosin. For this purpose, we used light microscopy (Olympus BX51, Tokyo, Japan). The necrotic zones were manually selected and the percentage of the necrotic area was determined using Cell v3.1 software, Olympus Soft Imaging Solutions (Münster, Germany).

### 2.4. Cell Culture and Viability Assay

Human liver-derived Hep G2 cells were obtained from the American Type Culture Collection (Manassas, VA, USA). The monolayer Hep G2 cell culture was trypsinized and the cell count was adjusted to 1.0 × 10^5^ cells/mL using DMEM containing 10% fetal bovine serum. Hep G2 cells were cultured in 24-multiwell culture plates at 2.5 × 10^4^ cells and were pretreated in the presence or absence of fraxin with a concentration of 1 to 100 μg/mL, or curcumin (20 μM) as a positive control, for 12 h, then the cells were followed by treatment with *t*-BHP for 12 h. Curcumin (20 μM) was used as a positive control. For determination of cell viability, cells were added with 200 μL of 5 mg/mL 3-[4,5-dimethylthiazol-2-yl]-2,5-diphenyltetrazolium bromide (MTT, Sigma, St. Louis, MO, USA) solution/well and were incubated further for 4 h in a humidified atmosphere (37 °C in 5% CO_2_). The medium was replaced with 1 mL dimethyl sulfoxide (DMSO). The absorbance was measured at 540 nm on a microplate reader (Molecular Devices Inc., Sunnyvale, CA, USA). Cell proliferation was expressed as percentage values in comparison with the negative PBS control, which was considered to represent 100% cell proliferation.

### 2.5. Measurement of ROS Generation

Cells were pretreated in the presence or absence of fraxin with concentrations of 1 to 100 μg/mL, or curcumin (20 μM) as a positive control, for 12 h, then the cells were followed by treatment with *t*-BHP for 12 h. After washing with PBS, the cells were stained with 10 μM 2′,7′-dichlorofluorescein diacetate in Hank’s balanced salt solution for 30 min in the dark. Subsequently, the cells were washed twice with PBS and were lysed with 1% Triton X-100 in PBS at 37 °C for 10 min. Fluorescence was measured at an excitation wavelength of 490 nm and an emission wavelength of 525 nm (Spectramax Gemini XS; Molecular Devices, Sunnyvale, CA, USA). Curcumin (20 μM) was used as a positive control.

Hydroxyl radical-scavenging assay was performed using the method described by Klouwen [17]. The reaction mixture was incubated at 37 °C for 30 min. Absorbance was measured at 520 nm using a UV-VIS spectrophotometer (Spectramax Gemini XS; Molecular Devices, Sunnyvale, CA, USA). The inhibition rate was calculated as follows: [(A1 − A2)/(A0 − A2)] × 100%, where A0 is the absorbance of the control, A1 is the absorbance of sample, and A2 is the absorbance of the blank sample. Vitamin C (50 μM) was used as a positive control.

### 2.6. ARE Luciferase Assay

The NAD(P)H dehydrogenase[quinone]1 (NQO1)–ARE luciferase construct, containing a three-tandem repeat of the ARE in the 5′-upstream region of NQO1, was introduced into the cells to determine transcriptional activation of Nrf2 by fraxin. Hep G2 cells were plated in 12-well plates overnight, serum-starved for 6 h, and then were transfected with luciferase construct and the pRL-SV plasmid (a plasmid that encodes for Renilla luciferase that is used to normalize transfection efficacy) in the presence of Lipofectamine^®^ 2000 (Invitrogen, San Diego, CA, USA) for 3 h. The activity of firefly luciferase was measured by adding Luciferase Assay Reagent II (Promega, Madison, WI, USA) according to the manufacturer’s instructions, and the Renilla luciferase reaction was initiated by adding Stop & Glo^®^ reagent (Promega). Relative luciferase activities were calculated by normalizing firefly luciferase activity with that of Renilla luciferase.

### 2.7. Nuclear Factor Erythroid-Derived 2-Related Factor 2 (Nrf2), Heme Oxygenase-1 (HO-1) Protein Expression Analysis

Hep G2 cells (2 × 10^6^ cells/well in six-well plate) were harvested by centrifugation at 200× *g* for 3 min. Cells were then washed with Tris-buffered saline (TBS; 20 mM Tris, pH 7.5, 130 mM NaCl) containing protease inhibitor and phosphatase inhibitor cocktails and placed on ice shortly after. Subsequently, cells were lysed by the addition of RIPA buffer directly to the dish. The nuclear/cytosol fractionation kit (Bio Vision Technology Inc., New Minas, NS, Canada) was used to separate nuclear and cytoplasmic proteins according to the manufacturer’s protocol. After isolation, protein concentration of the samples was determined using a micro BCA assay kit (Thermo Fisher Scientific, Waltham, MA, USA). Protein samples (20 µg) were separated on a 12% reducing SDS-PAGE and were transferred onto a nitrocellulose membrane. The membrane was blocked with 5% skim milk and was sequentially incubated with anti-Nrf2, anti-HO-1, or anti-GAPDH antibodies at 4 °C overnight. All antibodies were purchased from Cell Signaling (Danvers, MA, USA) and were used at 1:1000 dilution. Immunoreactive bands were visualized by horseradish peroxidase-conjugated secondary antibodies (1:1000 dilution, Enzo Life Sciences, Farmingdale, NY, USA) followed by ECL detection (Amersham Pharmacia Biotech, Piscataway, NJ, USA). Images were captured using a FluorChem E system (ProteinSimple, Santa Clara, CA, USA).

### 2.8. HO-1 Gene Expression Analysis

For reverse transcription polymerase chain reaction (RT-PCR), total RNA was extracted using a total RNA extraction kit (easy-BLUE, iNtRON Biotechnology, Sungnam, Korea). The RNA isolation protocol included a DNase I treatment step. RNA samples were quantified by measuring their OD260 values. All reaction mixtures contained 100 ng of RNA in a reaction volume of 25 μL. Primer and probe concentrations were 300 and 200 nM, respectively. Conditions for real-time quantitative RT-PCR were as follows: 30 min at 48 °C (RT, inactivation), 10 min at 95 °C (initial activation), then 40 cycles of amplification for 15 s at 95 °C (denaturation), and 1 min at 60 °C (annealing and extension). The primers and probes used for HO-1 (Hs01110250_m1) and GAPDH (Hs02758991_g1) amplification were obtained via TaqMan Gene Expression Assays (Applied Biosystems, Foster City, CA, USA). Data analysis was performed with SDS 2.1.1 Software (Applied Biosystems). Gene expression levels were normalized to the expression of GAPDH housekeeping gene. Relative e xpression level and PCR efficiency were evaluated [18].

### 2.9. Statistical Analysis

Data are expressed as the mean ± SD. Significant differences were compared using Student’s *t*-test. Statistical significance was defined as *p* < 0.05. All statistical analyses were performed using GraphPad Prism 5.0 software (Chicago, IL, USA).

## 3. Results

### 3.1. Protective Effect of Fraxin against CCl_4_-Induced Hepatic Damage

**Figure 2:**
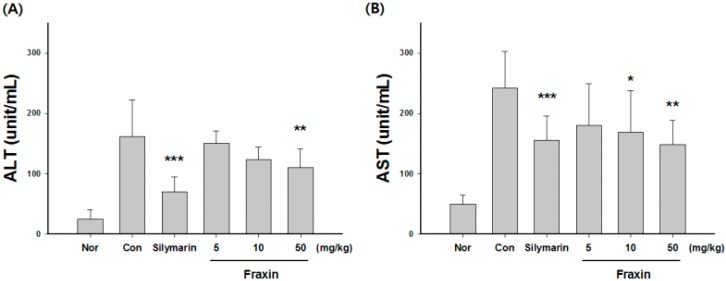
Effects of fraxin on serum alanine aminotransferase (ALT) (**A**) and aspartate aminotransferase (AST) (**B**) levels in carbon tetrachloride (CCl_4_)-treated rats. Rats were pretreated with fraxin or saline for five days, followed by a challenge with CCl_4_ (0.75 mL/kg, orally once) and blood was collected for serum enzyme determinations. The results are presented as the mean ± SD. Significant differences compared with the control group are indicated by * *p* < 0.05, ** *p* < 0.01 and *** *p* < 0.001.

**Figure 3:**
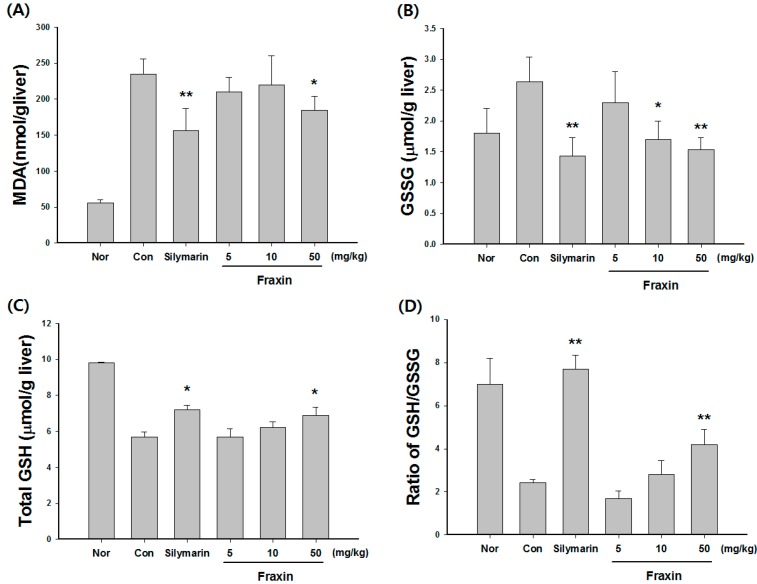
Effects of fraxin on hepatic (**A**) malondialdehyde (MDA); (**B**) oxidized glutathione (GSSG); (**C**) total glutathione (GSH); and (**D**) GSH/GSSH ratio level in carbon tetrachloride (CCl_4_)-treated rats. Rats were pretreated with fraxin or saline for five days followed by a challenge with CCl_4_ (0.75 mL/kg, orally once). The liver was excised and MDA, GSSG, total GSH, and the ratio of GSH/GSSG levels were determined as described in the Material and Methods section. The results are presented as the mean ± SD. Significant differences compared with the control group are indicated by * *p* < 0.05 and ** *p* < 0.01.

CCl_4_-treated rats showed a 5.2-fold and 4.9-fold increase in serum ALT (161.7 ± 60.7 units/mL) and AST (241.5 ± 61.1 units/mL) compared with the control group, respectively. Treatment with 50 mg/kg fraxin significantly blocked the CCl_4_-induced elevation of ALT (110.4 ± 30.4 units/mL) and AST (148.4 ± 40.4 units/mL); similar effects were observed in the silymarin-treated group (Figure 2). The MDA level increased by 4.1-fold in the CCl_4_-treated group (235.5 ± 42.1 nmol/g liver) compared with the control group (53.3 ± 17.2 nmol/g liver). However, treatment with 50 mg/kg fraxin significantly decreased the MDA level (150.5 ± 43.1 nmol/g liver; Figure 3A). The CCl_4_-treated group had significantly increased GSSG levels (2.6 ± 0.3 μmol/g liver) compared with the control group (1.8 ± 0.4 μmol/g liver). Treatment with 10 and 50 mg/kg fraxin significantly reduced the GSSG levels (1.7 ± 0.3 and 1.5 ± 0.2 nmol/g liver, respectively) compared with the GSSG levels of the CCl_4_-treated group (Figure 3B). Our results show that fraxin and silymarin-treated groups not only increase the level of total GSH, but also markedly improve the GSH/GSSG ratio (Figure 3C,D).

### 3.2. Histopathology of Hepatic Damage

**Figure 4:**
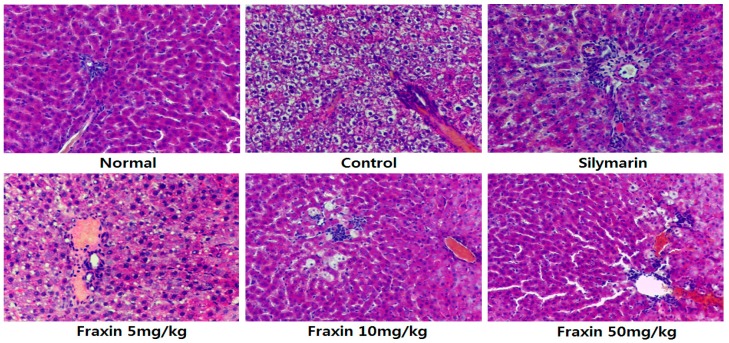
Histology images of liver tissues in rats with carbon tetrachloride (CCl_4_)-induced hepatic damage (200× magnification).

The liver biopsy with H and E staining is shown in Figure 4. CCl_4_ induced histopathological changes in the liver, with significant degeneration and necrosis of hepatocytes in the centrilobular region and perivenular inflammatory infiltrates. Sections from rats treated with CCl_4_ showed widespread areas of congestion and hemorrhage in the centrilobular zone, and necrosis involving many hepatocytes was observed in this area. Tissue sections from the 50 mg/kg fraxin group and silymarin appeared to provide greater protection from liver damage. In addition, a greater hepatoprotective effect was observed for the 50 mg/kg fraxin group, compared with the 10 mg/kg fraxin group.

### 3.3. Protective Effect of Fraxin against t-BHP-Induced Hepatotoxicity

**Figure 5:**
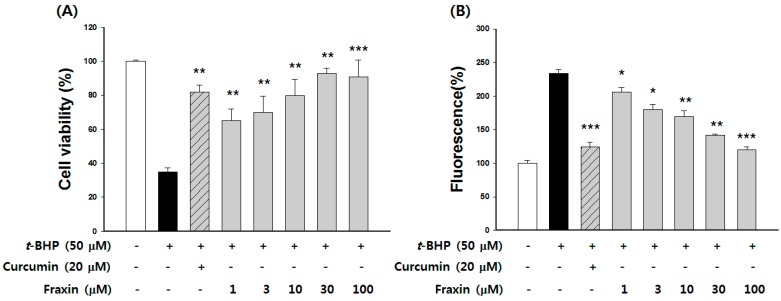
Effects of fraxin on (**A**) cytotoxicity and (**B**) inhibition of reactive oxygen species (ROS) generation in *tert*-butyl hydroperoxide (*t*-BHP)-treated cells. Cells were treated with fraxin and subsequently incubated for 12 h with *t*-BHP (50 µM). Cell viability and ROS generation were determined as described in the Material and Methods section. Curcumin was used as a positive control. The results are presented as the mean ± SD. Significant differences compared with the *t*-BHP-treated group are indicated by * *p* < 0.05, ** *p* < 0.01 and *** *p* < 0.001.

**Figure 6:**
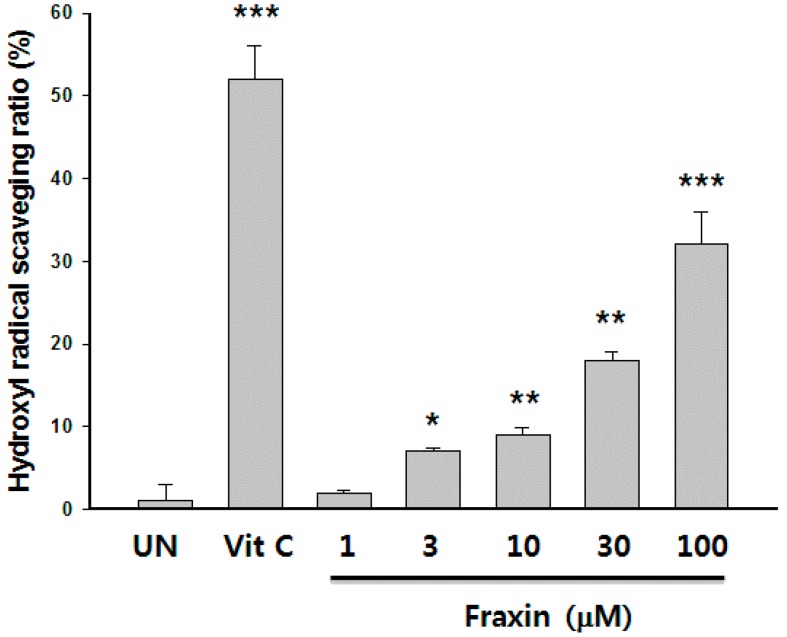
Hydroxyl radical-scavenging effect of fraxin. Vitamin C was used as a positive control. The results are presented as the mean ± SD. Significant differences compared with the untreated group are indicated by * *p* < 0.05, ** *p* < 0.01 and *** *p* < 0.001.

MTT assay showed that the highest tested concentration (100 μM) of fraxin was non-cytotoxic on Hep G2 cells (data not shown). Curcumin was used as a positive control and showed significant cytoprotective effects and ROS scavenging activity. Treatment with *t*-BHP inhibited Hep G2 cell growth by 66% compared with the untreated cells, whereas the addition of fraxin at non-cytotoxic concentrations caused a significant dose-dependent improvement in cell viability (Figure 5). *t*-BHP stimulation increased the ROS generation by 124%. The addition of fraxin at non-cytotoxic concentrations significantly decreased the *t*-BHP-induced ROS generation in a dose-dependent manner (Figure 5B). The hydroxyl radical-scavenging activity of fraxin was compared with that of a representative antioxidant, vitamin C. Hydroxyl radical-scavenging activity increased from 1.93% to 32.0% when treated with fraxin concentrations of 1–100 μM compared with the untreated group. Hydroxyl radical-scavenging activity of 100 μg/mL fraxin was similar to that of 50 µM vitamin C (Figure 6).

### 3.4. Effect of Fraxin on the Nrf2 Activation and Target Gene Induction

**Figure 7:**
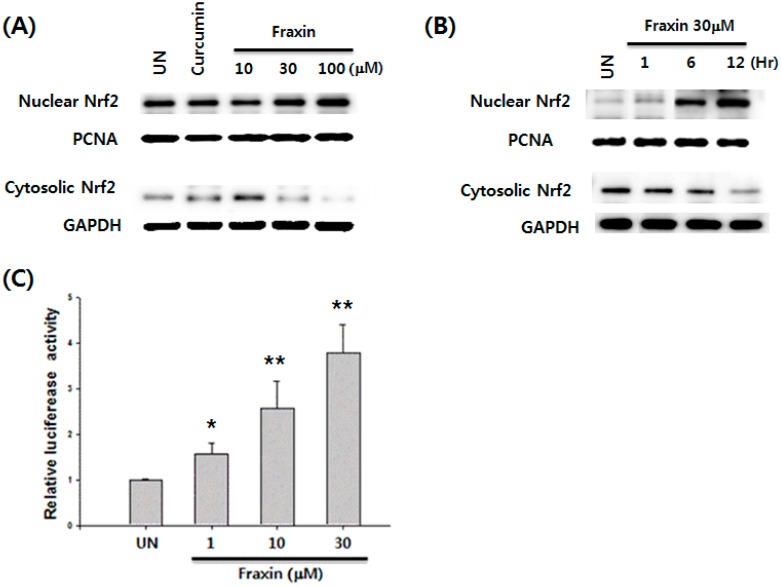
Effect of fraxin on nuclear factor erythroid-derived 2-related factor 2 (Nrf2) activation in Hep G2 cells. (**A**) Cells were treated with indicated concentrations of fraxin for 12 h, and Nrf2 translocation was determined; (**B**) Time course of nuclear Nrf2 translocation in Hep G2 cells treated with 30 µM fraxin; (**C**) Cells were exposed to 1–30 µM fraxin for 12 h, and Nrf2 transactivation was determined by ARE luciferase activity. The results are presented as mean ± SD. Significant differences compared with the untreated group are indicated by * *p* < 0.05 and ** *p* < 0.01.

The effect of non-cytotoxic concentrations (1–100 µM) of fraxin on the translocation of Nrf2 protein was examined in Hep G2 cells. As shown in Figure 7A, fraxin caused a dose-dependent increase in Nrf2 protein levels. The nuclear fractions of fraxin-treated cells showed a gradual increase in Nrf2 levels, whereas Nrf2 levels concomitantly decreased in the cytoplasmic fractions. As shown in Figure 7B, fraxin also caused a time-dependent increase in Nrf2 protein levels. At a fraxin concentration of 30 µM, an increase in the Nrf2 expression was observed after 6 h of treatment. We then examined reporter gene analysis using an ARE as a reporter to verify fraxin-induced ARE activation. ARE luciferase constructs that contain three-tandem repeats of ARE in the 5′-upstream region of NQO1 were transfected into Hep G2 cells to examine transactivation by fraxin. Exposure of the transfected cells to fraxin resulted in a significant increase in luciferase activity of the NQO1–ARE reporter construct (Figure 7C).

**Figure 8:**
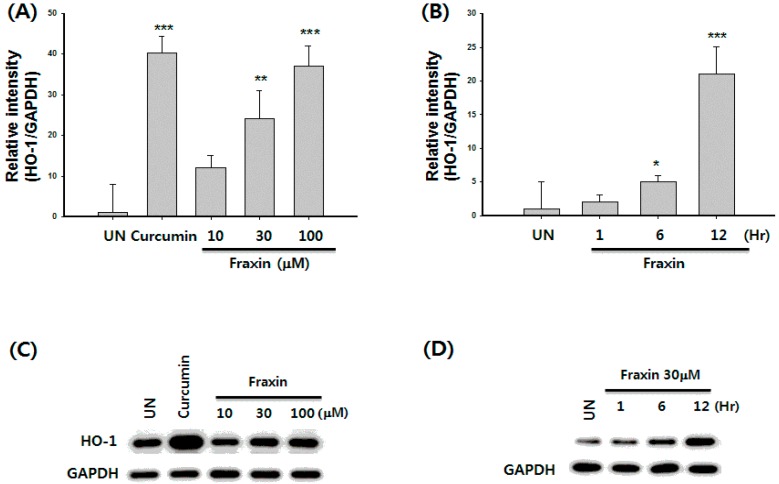
Effects of fraxin on expression of heme oxygenase 1 (HO-1) messenger RNA (mRNA) and protein in Hep G2 cells. Cells were incubated for 12 h with the indicated concentrations of fraxin (**A**,**C**) or for the indicated times with 30 µM fraxin (**B**,**D**). Significant differences compared with the untreated group are indicated by * *p* < 0.05, ** *p* < 0.01 and *** *p* < 0.001.

Nuclear translocation of activated Nrf2 is an important upstream regulator for HO-1 expression. Therefore, we investigated whether treatment with fraxin induced the expression of HO-1 in Hep G2 cells. Curcumin, an HO-1 inducer, was used at 20 µM as a positive control. Fraxin-induced HO-1 mRNA expression consistently increased in a dose-dependent manner, as assessed by RT-PCR analysis (Figure 8A). An increase in HO-1 mRNA expression was observed after 6 h of treatment with 30 µM fraxin (Figure 8B). As shown in Figure 8C, fraxin increased HO-1 protein levels in a dose-dependent manner. Treatment with 100 µM fraxin showed maximum HO-1 expression. At fraxin concentration of 30 µM, HO-1 expression started to increase after 6 h treatment (Figure 8D).

## 4. Discussion

Several studies have suggested that the antioxidant activity against free radicals may be an important mechanism of hepatoprotection [20]. Many natural products containing flavonoids, coumarins, and polyols have been investigated for the development as antioxidants [4,15,21,22,23].

Coumarin derivatives have recently gained a great amount of attention because of their broad pharmacological activities. Fraxin (7-hydroxy-6-methoxycoumarin 8-glucoside) has been isolated from the bark of *F. ornus* (Oleaceae), *A. hippocastanum* (Sapindaceae), as well as *A. tegmentosum* [9,11]. Several published studies have demonstrated its antioxidant effects [11,13,24]. Previous studies also suggested that fraxin isolated from various plants showed various activities [13,25,26,27]. Fraxin from *F. excelsior* (Oleaceae) had anti-inflammatory and antimetastatic properties, the former probably because of its direct action on cells, predominantly on macrophages inhibitory effect on 5-HETE production [28]. Fraxin from *F. ornus* (Oleaceae) is also known to act as a choleretic agent for stimulating bile flow and aiding digestion, and has noted activity for preventing the development of abnormal growths [29]. However, hepatoprotective activities of fraxin remain to be elucidated. Therefore, we investigated the hepatoprotective properties and the underlying mechanisms of the fraxin’s activity.

CCl_4_ is used to induce liver toxicity to allow the testing of drugs for their hepatoprotective activities. Silymarin is used as standard hepatoprotective compound since it is reported to have a protective effect on the hepatocytes [4,14]. In the present study, we also used CCl_4_ to induce hepatotoxicity in rats. In the CCl_4_-treated group, the ALT and AST levels dramatically increased, indicating severe hepatocellular damage [30,31]. Significantly suppressed ALT and AST levels were observed after treatment with fraxin. The hepatoprotective effect of fraxin appeared to be similar to that of silymarin, a potent hepatoprotective agent. An increase in MDA levels, as seen in the present study after CCl_4_ administration, indicates increased lipid peroxidation, leading to tissue damage and failure of antioxidant defense mechanisms required to prevent the formation of excessive free radicals [30,32,33]. Significant reduction in the hepatic lipid peroxidation was observed in the group treated with 50 mg/kg fraxin. Changes in MDA levels and serum AST and ALT levels displayed the same trends. GSH is an important scavenger molecule that protects against oxidative stress in the liver. Its depletion in hepatocytes could endanger the antioxidant defense system, leading to accumulation of ROS. Previous studies have shown that CCl_4_ decreased the activities of antioxidant enzymes [14,22].

Measurement of glutathione (GSH) and glutathione disulfide (GSSG) separately, and related intermediates, are important in assessing the redox and metabolic status of biological systems in vivo and in vitro. Measurements of both GSH and GSSG are useful in experimental systems because changes in the GSH/GSSG ratio are associated with human disease, aging, and cell signaling events [18,34]. In this study, total GSH and GSH/GSSG ratio levels in the livers of CCl_4_-treated group rats were significantly lower than those in the control group, suggesting that the decrease in antioxidant scavenging capacity in the CCl_4_-treated group occurred due to severe stress injury. Fraxin markedly elevated the levels of total GSH and GSH/GSSG ratio, indicating that inhibition of the oxidative stress cascade was one of the main mechanisms involved in CCl_4_-induced hepatic damage.

Lipid peroxidation can be assessed by the measurement of MDA, 4-HNE, F2-isoprostanes, conjugated dienes, and ethane and pentane gases. The quantification of F2-IsoPs in urine and plasma is most convenient and least invasive [35]. MDA is a frequently used biomarker that is measured in plasma and hepatic tissue as a thiobarbituric acid-reactive (TBAR) material [14,36]. Although TBAR assay has been extensively criticized as being non-specific for MDA, it remains widely used in hepatic tissue. Our study measured MDA for a comprehensive survey of oxidative stress in hepatic tissue. Administration of CCl_4_ has been reported to elevate levels of MDA, a product of lipid peroxidation in the liver of rats. Previous studies have attributed the increase in MDA levels to enhanced lipid peroxidation. This leads to tissue damage and failure of antioxidant defense mechanisms required to prevent the formation of excessive free radicals. The results of this study showed a significant increase in MDA levels in the CCl_4_-treated rats compared with the control group. However, fraxin treatment significantly decreased the CCl_4_-induced increase in the lipid peroxidation, indicating that the hepatoprotective effect of fraxin on CCl_4_-induced liver injury involved the alleviation of lipid peroxidation.

Histopathology is an important clinical standard for the diagnosis of hepatic damage. In addition, histopathological examination of rat liver sections has been reported as an effective method for evaluation of hepatoprotective activity in a CCl_4_-induced rat hepatic damage model. From the sections examined, fraxin exerted a preventive effect against CCl_4_ induced hepatic damage. Fraxin showed dose-dependent radical-scavenging activities in the present study. Lin et al. [24] reported that fraxin showed antioxidant activity against DPPH with an IC_50_ value of 40.5 µg/mL. Results of the present study also suggested that fraxin is a potent antioxidant.

We used *t*-BHP to induce hepatotoxicity in Hep G2 cells. The cell viability and inhibition of ROS generation was then examined in vitro. Curcumin, the major biologically active phenolic compound from *Curcuma longa* with strong antioxidant and hepatoprotective activities, was used as a positive control [35]. Treatment with curcumin showed significant cytoprotective and ROS scavenging activities. All concentrations of fraxin significantly protected against cell death and inhibited the ROS production in a dose-dependent manner. The cytoprotective properties of antioxidants have been partially attributed to their ability to induce cytoprotective enzymes [33].

To understand the mechanism of hepatoprotection, the effect of fraxin on the activation of the Nrf2/HO-1 pathway was examined. HO-1, one of the most important cytoprotective enzymes, is known to have a putative role in several different models of hepatic injury. This inducible enzyme catalyzes the rate-limiting step of free heme degradation into free iron, carbon monoxide, and biliverdin, the last of which is subsequently catabolized into bilirubin, a potent endogenous antioxidant. Previous studies showed that the induction of HO-1 expression was required to suppress *t*-BHP-induced ROS generation [30,32]. The present study showed that fraxin induced HO-1 protein and mRNA expression in Hep G2 cells in a dose- and time-dependent manner.

Nrf2 is a key transcription factor, which plays a central role in cellular defense against oxidative stress by inducing the expression of cytoprotective and phase-2 detoxifying genes. Recent studies have suggested that phytochemicals can activate Nrf2 by directly binding to the Keap1 protein through covalent linkages, resulting in the induction of cytoprotective proteins, including HO-1 [30,36]. This study showed that fraxin significantly increased Nrf2 levels and efficiently promoted the translocation of Nrf2 into the nucleus in Hep G2 cells. These results are in agreement with those of previous studies, which indicate that phytochemicals exert hepatoprotective effects via HO-1 induction.
